# Olfactory Stimulation by Fennel (*Foeniculum vulgare* Mill.) Essential Oil Improves Lipid Metabolism and Metabolic Disorders in High Fat-Induced Obese Rats

**DOI:** 10.3390/nu14040741

**Published:** 2022-02-10

**Authors:** Seong Jun Hong, Sojeong Yoon, Seong Min Jo, Hyangyeon Jeong, Moon Yeon Youn, Young Jun Kim, Jae Kyeom Kim, Eui-Cheol Shin

**Affiliations:** 1 Department of Food Science, Gyeongsang National University, Jinju 52725, Korea; 01028287383a@gmail.com (S.J.H.); dbsthwjd0126@naver.com (S.Y.); jojo9875@naver.com (S.M.J.); giddus9967@naver.com (H.J.); ringspot@naver.com (M.Y.Y.); 2 Department of Food and Biotechnology, Korea University, Sejong 30019, Korea; yk46@korea.ac.kr; 3 Department of Behavioral Health and Nutrition, University of Delaware, Newark, DE 19716, USA; jkkim@udel.edu; 4 Department of GreenBio Science, Gyeongsang National University, Jinju 52725, Korea

**Keywords:** *Foeniculum vulgare* Mill., fennel essential oil, metabolic health, obesity

## Abstract

In this study, odor components were analyzed using gas chromatography/mass spectrometry (GC/MS) and solid-phase microextraction (SPME), and odor-active compounds (OACs) were identified using GC-olfactometry (GC-O). Among the volatile compounds identified through GC-O, *p*-anisaldehyde, limonene, estragole, anethole, and trans-anethole elicit the fennel odor. In particular, *trans*-anethole showed the highest odor intensity and content. Changes in body weight during the experimental period showed decreasing values of fennel essential oil (FEO)-inhaled groups, with both body fat and visceral fat showing decreased levels. An improvement in the body’s lipid metabolism was observed, as indicated by the increased levels of cholesterol and triglycerides and decreased levels of insulin in the FEO-inhaled groups compared to group H. Furthermore, the reduction in systolic blood pressure and pulse through the inhalation of FEO was confirmed. Our results indicated that FEO inhalation affected certain lipid metabolisms and cardiovascular health, which are obesity-related dysfunction indicators. Accordingly, this study can provide basic research data for further research as to protective applications of FEO, as well as their volatile profiles.

## 1. Introduction

Obesity is a non-communicable disease that can induce complications, and has been recognized as a serious health problem worldwide [1]. Obesity, defined as the state of accumulation of energy and fat in the body, is caused by an increase in energy intake and a decrease in energy expenditure. This status is defined as abnormal fat accumulation [2,3]. Increased body fat can lead to leptin resistance, a status in which the level of leptin increases, while there is no variation in food intake. In addition, this status can increase the prevalence of type 1 and type 2 diabetes, and it can lead to hypertension due to an increase in blood pressure and heart rate beyond the normal status [1,3,4]. Furthermore, excessive accumulation of body fat may induce metabolic syndrome via an increased prevalence of cardiovascular diseases, which may be caused by increased levels of total cholesterol (TC), low-density lipoprotein cholesterol (LDL-cholesterol), triglyceride (TG), and decreased high-density lipoprotein cholesterol (HDL-cholesterol) [1,3]. Many researchers have reported various functional materials and substances that have been investigated for decreasing body weight and increasing energy expenditure [1,2,5,6].

A recent study reported that the development of natural functional materials has been of increasing interest to undermine the side-effects of obesity [2]. Functional foods, which are made of natural functional materials, have been reported to reduce obesity and metabolic syndrome. In addition, functional food hardly ever has side effects, unlike synthetic medicine for obesity [2,6]. Many studies on the physiological activity of functional materials and medicinal herbs have been reported, including a study on the suppression of white adipose tissue accumulation using pine bark extracts [5], anti-obesity via inhalation of citronella essential oil [7], and anti-obesity and metabolic health via inhalation of patchouli essential oil (PEO) [1].

Fennel (*Foeniculum vulgare* Mill.) originated in southern Europe and the Mediterranean, and is now a herbaceous and perennial plant mainly cultivated in China. In addition, all of its parts can be used, such as its bulbous roots, stem, and seeds. Owing to its strong scent, fennel is used in flavoring agents, medicines, cosmetics, and in the food industry [8]. Recent studies have reported that fennel and fennel essential oil (FEO) may play a role in the control of the central nervous system (CNS) and autonomic nervous system (ANS) in the body [1,8,9]. Furthermore, fennel has been demonstrated as a medicinal herb with pharmacological and physiological activities, such as suppression of food intake, body weight, white adipose tissue, blood glucose, and cardiovascular diseases [8,9,10,11]. 

Volatile compounds in FEO can be influenced by agro-environmental conditions (climatic conditions). Thus, FEO, which was cultivated from five different agro-environmental conditions in India, represented that the concentrations of limonene in FEO cultivated from Moradabad (latitude: 28°3′ N; longitude: 78°77′ E), Tanakpur (latitude: 29°07′ N; longitude: 80°10′ E), and Haldwani (latitude: 29°13′N; longitude: 79°31′ E) detected more than 30% of FEO; however, the concentrations of limonene in FEO cultivated from Pithoragarh (latitude: 30°08′ N; longitude: 80°36′ E) and Didihat (latitude: 29°80′ N; longitude: 80°24′ E) detected less than 10% of FEO. Additionally, the concentrations of *trans*-anethole in FEO cultivated from Moradabad and Haldwani detected less than 30% of FEO, while concentrations of *trans*-anethole in FEO cultivated from Tanakpur, Pithoragarh, and Didihat detected more than 40% of FEO [12]. Furthermore, volatile profiles of fennel in Spain have been identified where the concentrations of volatile compounds were varied according to the agor-environmental conditions [13]. 

Although several previous studies of fennel intake have been reported, research investigating the mechanism and observation of metabolic health in vivo after FEO inhalation is insufficient. In the present study, we investigated the changes in metabolic aspects, such as body weight, food intake, hormones, blood pressure, heart rate, and blood glucose in vivo in Sprague Dawley rats by controlling the FEO concentration and inhalation period. 

## 2. Materials and Methods

### 2.1. Essential Oil

Fennel essential oil (FEO) was purchased from Aroma Care Solution (Helga Stolz GmbH Co., Grafenwoerth, Austria) and stored at 4 °C before the experiment to minimize any biological activity and chemical composition of the sample.

### 2.2. Volatile Compounds and Odor Description

Volatile compounds of FEO were analyzed by headspace analysis using polydimethylsiloxane (PDMS) coated with 100 µm. One gram of sample was placed in a collection bottle, sealed with an aluminum cap, and exposed to the headspace of the heated sample at 60 °C. Volatiles, which are absorbed by the SPME fiber, were analyzed using gas chromatography-mass spectrometry (GC-MS; Agilent 7890A & 5975C, Agilent Technologies, Santa Clara, CA, USA) and a HP-5MS column (30 m × 0.25 mm i.d. × 0.25 μm film thickness). The analysis was done in an oven that was first kept at a temperature of 40 °C for 5 min, and then heated to 200 °C at a speed of 5 °C. The injector temperature was 220 °C; the flow rate of the carrier gas helium was 1.0 mL/min; and the split ratio was splitless. Each compound separated from the total chromatogram (TIC) was sympathetic to the mass spectrum library (NIST 12) and literature, and each concentration of volatile compound was calculated by converting the peak area of the internal standard material (pentadecane) into a peak area of g/100 g. During volatile analysis, the odor description of each compound was measured using GC-MS, and the four levels of odor intensity and the duration of scent were measured using GC-olfactometry (GC-O) [1].

### 2.3. Animal Care and Experimental Design

This study was conducted with the approval of Document #: IACUC-4 of Gyeongnam National University of Science and Technology in accordance with the Animal Protection Act. In the present study, a total of 24 experimental animals, which were four-week-old male Sprague Dawley rats, were purchased from Coretec Co. (Busan, Korea). After a week of preliminary adaptation, the experimental animals were randomly separated into four groups and bred for 12 weeks. In detail, animals were randomly separated into groups: the control group was fed a normal diet (N; *n* = 6), and the control group was fed a 45% high-fat diet (H; *n* = 6). Both groups *n* and H were given 30 min/day inhalation of distilled water (DW) at the same time per day for 12 weeks. The low-dose inhaled FEO group (H-LFI; *n* = 6) with a high-fat diet (HFD) was given 30 min/day inhalation of 0.3% FEO for 12 weeks, and the high-dose inhaled FEO group (H-HFI; *n* = 6) with HFD was given 30 min/day inhalation of 1% FEO for 12 weeks. The body weights, food intake, and length of experimental animals were measured at a fixed time each week. After fasting for 16 h before dissection, blood was collected using a syringe containing 20 mg ethylenediaminetetraacetic acid. Subsequently, the aorta was cut, and the rats were sacrificed. The collected blood samples were stored on ice for 30 min and then centrifuged at 1008 G to separate the plasma. Next, the brain, heart, liver, kidney, white adipose tissue (WAT), brown adipose tissue (BAT), lung, adrenal glands, spleen, testis, and epididymis were extracted, and organs and plasma were stored at −80 °C [1].

### 2.4. Body Composition Assessment Using Dual-Energy X-ray Absorptiometry (DXA)

The body composition of rats was measured using InAlzer (Medikors Co., Seongnam, Korea) at the 12th week after fasting for 16 h. Before analyzing body composition, the rats were anesthetized with isoflurane using XGI-8 (Caliper Life Science Co., Hopkinton, MA, USA). Subsequently, body composition was measured for lean body mass, body fat mass, body fat percentage, and bone mineral density (BMD), and the analysis was conducted without dissection processing. 

### 2.5. Plasma Biomarker Analysis

Plasma concentrations of TG, TC, HDL-cholesterol, aspartate transaminase (AST), and alanine transaminase (ALT) were analyzed using commercial kits (Asan Pharm Co., Seoul, Korea). Plasma insulin and leptin levels were analyzed using an ELISA kit (Bertin Technologies Co., Montigny le Bretonneux, France). Plasma cortisol and testosterone levels were analyzed using a hormone ELISA kit (Mybio Source Inc., San Diego, CA, USA).

### 2.6. Blood Glucose Assessment

The blood glucose levels in rats were analyzed after fasting for 16 h using a glucometer (Accu-Chek, Roche Diagnostic, Basel, Switzerland) and were measured at the 1st, 7th, and 12th weeks, respectively. For blood glucose analysis, blood was collected from the tail vein of rats. The experiments were performed in duplicate for each animal, and three measurements with less deviation, excluding the highest and lowest values, were calculated and recorded as the mean and standard deviation in each group [14].

### 2.7. Blood Pressure Assessment

Changes in the blood pressure and pulse of experimental animals were analyzed using an animal blood pressure meter (BP-2000, Visitech Systems Co., Apex, NC, USA), and the measurements were made on the tail of experimental animals at a fixed time in the 1st and 12th weeks. Blood pressure and pulse were measured 30 times per rat, including 10 preliminary tests and 20 main tests, and six measured values with small deviations, excluding the highest and lowest values, were calculated and recorded as the mean and standard deviation [1].

### 2.8. Statistical Analysis

In this study, the experiments were conducted in duplicate or triplicate, from which the standard deviations (SD) and mean were identified and used to present the results. Each group was non-parametrically compared using the Friedman test with the chi-square distribution, following Dunn’s post-test. *P*-values less than 0.05 were considered statistically significant (SAS Institute Inc., Cary, NC, USA).

## 3. Results

### 3.1. Volatile Compounds

**Figure 1:**
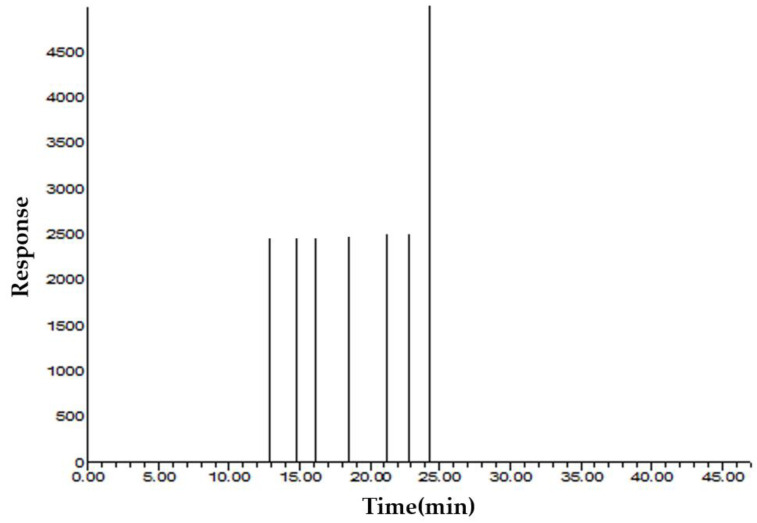
Representative aroma gram of odor active compounds (OACs) in fennel (*Foeniculum vulgare* Mill.) essential oil by GC-O test.

The results of volatile compounds in FEO using GC/MS and GC-O are shown in Table 1 and Figure 1. A total of seven odor-active compounds (OACs), including one aldehyde, five hydrocarbons, and one ketone, were detected. Among the volatiles detected by GC/MS, p-anisaldehyde, limonene, estragole, cis-anethole, and trans-anethole elicit fennel-like odors, which were detected by GC-O, whereas a-pinene and fenchone elicit herb and fragrance-like odors, which were detected by GC-O. Trans-anethole and fenchone had the highest and second-highest concentrations among FEO volatiles, respectively, and both trans-anethole and cis-anethole showed the highest odor intensity [2]. 

Among fennel volatiles, trans-anethole, fenchone, and estragole, which are described as major volatiles of fennel, are known to have higher concentrations and odor activity than the other fennel volatiles, as detected by GC-O [13]. Several studies have reported trans-anethole and isomers cis-anethole and estragole as major fennel volatiles [13,15,16]. A previous study reported that volatiles in FEO were extracted from hydrodistillation, supercritical fluid extraction, and microwave extraction methods. Anethole had the most abundant content of FEO (<50%), and fenchone had the second most abundant content (approximately 20%) [17]. On the other hand, another study reported that volatiles in FEO, which were extracted from ultrasound-assisted extraction, showed the concentration of anethole more than 80% among all volatiles, and the concentration of fenchone showed to be less than 10% among all volatiles [18]. Trans-anethole, cis-anethole, and estragole elicit fennel odors and have anti-obesity, anti-cholesterol, anti-inflammatory, and anti-stress effects [15]. Fenchone has antifungal, antidepressant, local anesthesia, and wound healing effects [16]. α-Pinene, a monoterpene compound, is found in several medicinal herbs and exerts positive effects by suppressing tumors, oxidation, bacteria, inflammation, stress, convulsion, and acute pain [19]. Limonene, another major monoterpene compound, is known for its anti-tumor and anti-inflammatory effects and exerts positive effects by reducing hepatotoxicity through reduced AST and ALT levels [16]. A previous study reported that limonene is a major volatile component of grapefruit oil and lowers blood glucose levels via inhalation [20]. The above studies on the pharmacological effects of volatiles show that FEO may exert positive physiological effects in vivo via inhalation.

### 3.2. Food Intake, Body Weight, Body Composition, and Length

**Figure 2:**
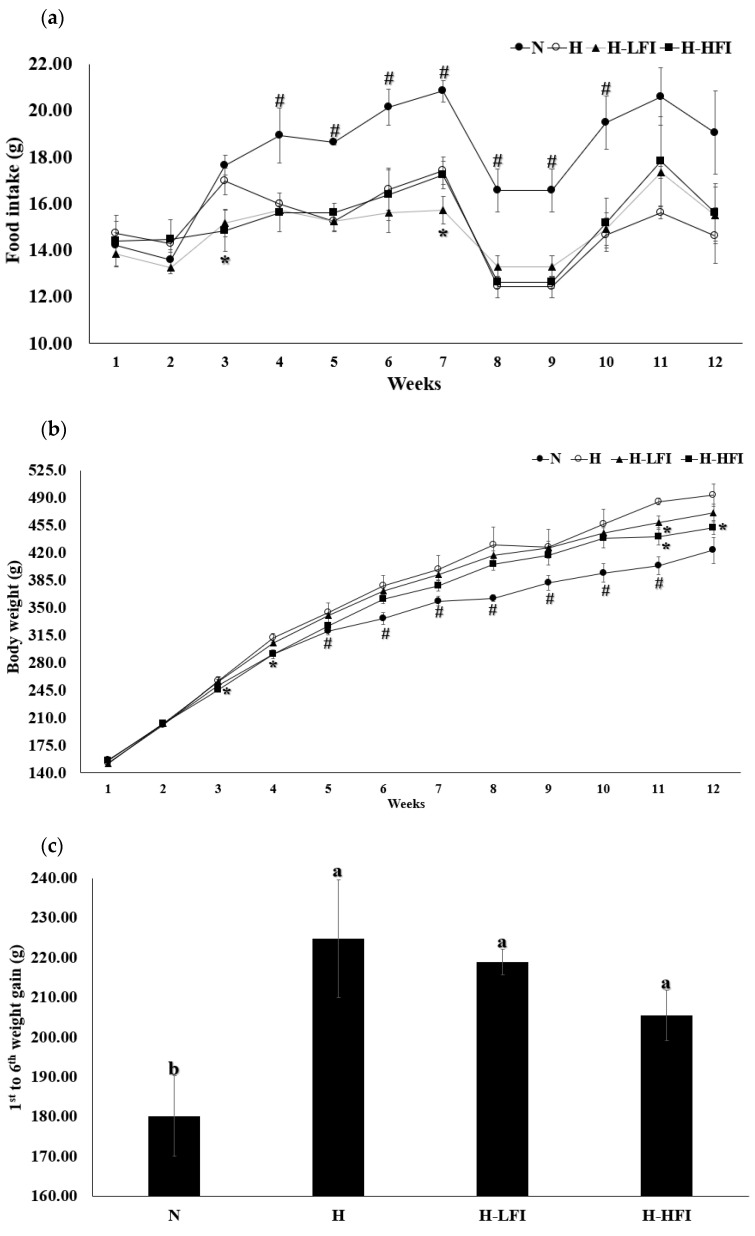

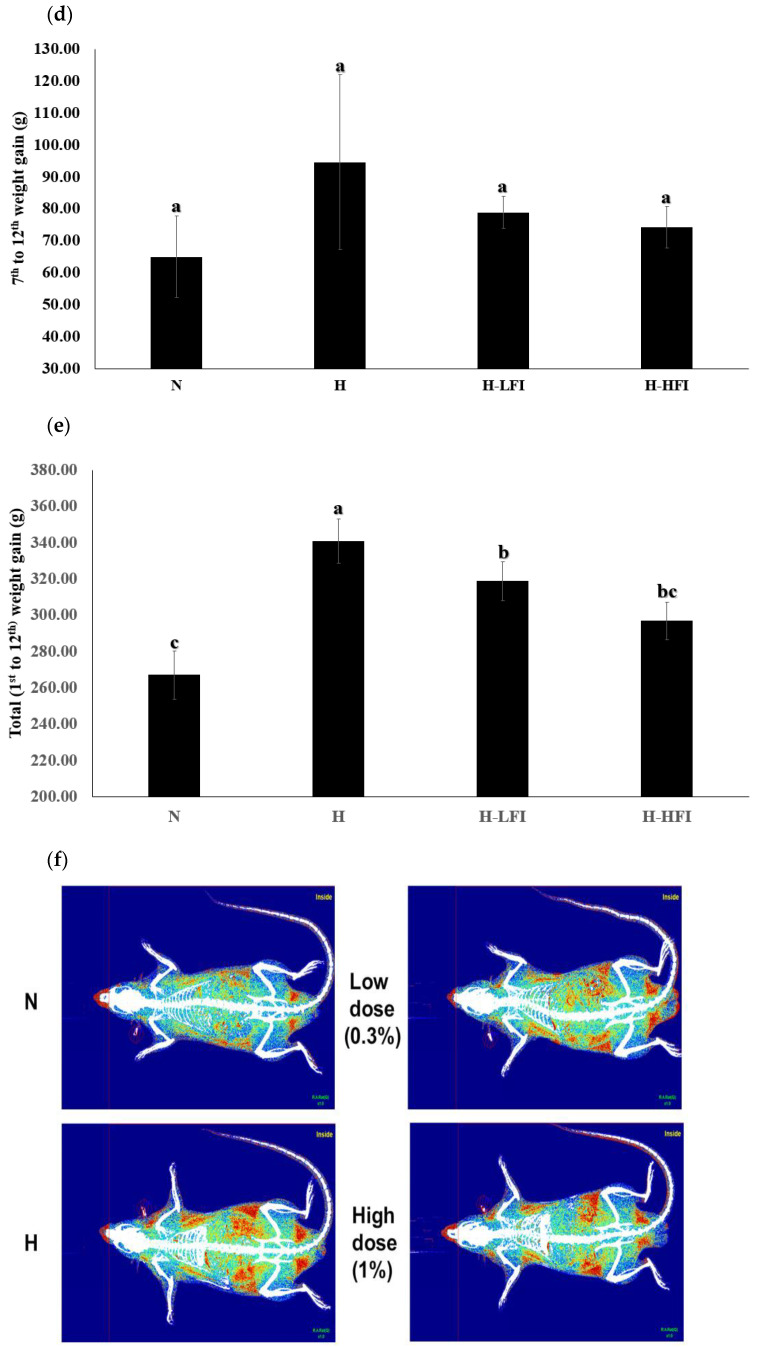
Measurement of blood, food intake, weight, and body composition in rats. (**a**) Change of food intake. (**b**) Change of body weight. (**c**) Comparisons of body weight gain during the period from the first to the seventh week. (**d**) Comparisons of body weight gain during the period from the 7th to the 12th week. (**e**) Comparisons of body weight gain during the period from the 1st to the 12th week. (**f**) Analysis of body composition on rats using the dual-energy X-ray absorptiometry (DXA) system (White—Bone/Blue—Lean/Red—Fat). The data are presented as the means ± SD values, # *p* < 0.05, comparison among all groups. * *p* < 0.05, compared with the high-fat diet (HFD) group. Means with different letters (a–c) corresponds to the significant differences determined through the non-parametric Friedman test followed by Dunn’s test (*p* < 0.05).

Changes in food intake, average daily food intake, and average daily calorie intake were measured during the experimental period (12 weeks), and the results are presented in Table 2 and Figure 2a. During FEO inhalation, the H-LFI and H-HFI groups showed no appearance or behavioral abnormalities. The food intake of group N, which was fed a normal diet, was higher than that of the other groups (*p* < 0.05). The food intake of groups H and H-LFI showed no significant differences among the high-fat diet (HFD)-fed groups. However, the H-HFI group showed decreased weeks (fourth and seventh weeks) of food intake compared to groups H and H-LFI (*p* < 0.05). In contrast, H-HFI showed no significant differences among the HFD-fed groups. Unlike food intake, the calorie intake of group N was the lowest compared to the other groups (*p* < 0.05), with no significant differences among all HFD-fed groups. 

Changes in body weight were measured every week, and the results are shown in Figure 2b. Body weight and weight gain in group N showed the lowest variation compared to the other groups during the experimental period (12 weeks). In the 11th week, group H-LFI had significantly lower body weight than group H, and the H-HFI group had significantly lower body weight than group H in the 3rd, 4th, 11th, and 12th weeks (*p* < 0.05). There were no significant differences in average weight gain among the HFD-fed groups from the 1st week to the 6th week, as well as from the 7th week to the 12th week. On the other hand, the FEO-inhaled groups (H-LFI and H-HFI) had significantly lower average weight gain compared to group H from the 1st week to the 12th week (*p* < 0.05). In particular, the average weight gain of group H-HFI did not differ significantly from that of group N from the 1st week to the 12th week (*p* > 0.05). The food efficiency ratio (FER) of group N was the highest compared to the other groups (*p* < 0.05), and the FER of the FEO-inhaled groups did not differ significantly from that of group H.

Body composition was measured using the DXA system, and the results are shown in Figure 2f. The ratio of fat-free mass did not differ significantly among all groups; however, the FEO-inhaled groups showed a relatively increased ratio compared to group H. Unlike fat-free mass, the ratio of body fat increased in group H compared to group N (*p* < 0.05); the H-LFI and H-HFI groups showed a lower body fat ratio compared to group H (*p* < 0.05). Regardless of FEO inhalation and types of food intake, bone mineral density (BMD), bone area, and bone volume did not differ significantly among the groups. 

Changes in the length of experimental animals were analyzed and are presented in Table 2. All animal groups showed an increase in length during the breeding period (12 weeks). In the first week, the length of the animals in group N were the highest compared to those in the other groups (*p* < 0.05); however, the FEO-inhaled groups (H-LFI and H-HFI) were significantly longer than those in groups N and H in the 12th week (*p* < 0.05). In addition, length gain increased in the FEO-inhaled groups compared to the DW-inhaled groups (*p* < 0.05). The body mass index (BMI) was higher in group H than in group N (*p* < 0.05). H-LFI and H-HFI were lower than H (*p* < 0.05). In particular, the FEO-inhaled groups did not differ significantly from group N (*p* < 0.05).

Fennel regulates food intake and causes decreased body weight by reducing food intake [21,22]. In the present study, the inhalation of high-dose FEO by group H-HFI led to the identification of the week of decreased trends of food intake; however, the daily average food intake did not decrease for 12 weeks (Table 2 and Figure 2). Bae et al. reported that the intake of fennel decreased appetite in overweight female subjects [9]. In addition, Heo et al. reported that the FEO-inhaled group had a relatively decreased food intake in rats, but with no significant difference from the control group [22]. According to previous studies, fennel inhalation may have a lower regulatory effect on food intake than on fennel intake. Weight gain is associated with changes in food intake [21]. In the present study, increased body weight in HFD-fed groups may have been associated with calorie intake (Table 2). Changes in body weight were hardly influenced by changes in food intake; however, FEO inhalation influenced the reduction in body fat mass and body weight. A previous study reported that body weight was lower in the FEO-inhaled group than in the control group (*p* < 0.05), which was similar to our results [22]. Moreover, a previous study reported that the intake of fennel affected decreases in body weight compared to the non-intake of fennel [11]. In addition, another study reported that trans-anethole, a major compound of fennel, increased the activation of AMP-activated protein kinase (AMPK) and peroxisome proliferator-activated receptor α (PPARα) in adipocytes, thereby decreasing body fat accumulation and increasing body weight [23]. In this study, reduction of body weight was associated with body fat, and the anti-obesity effect may be influenced by inhalation of FEO.

The BMI is an index of length and body weight [21]. In this study, a reduction in BMI was related to a reduction in body weight and increase in length. A previous study identified that the growth of length was influenced by the inhalation and concentration of PEO [1]; therefore, the inhalation of FEO may also be one of the various factors related to the growth of length. 

### 3.3. Organ Weight

The weights of 11 organs were measured after dissection in the experimental animals (Table 2). The brain weights ranged from 3.57 ± 0.25 to 3.98 ± 0.39 g/kg, while the liver weights ranged 24.44 ± 0.64 to 25.41 ± 2.28 g/kg. The heart weights ranged from 4.46 ± 0.11 to 5.71 ± 0.18 g/kg, while the kidney weights ranged from 2.34 ± 0.12 to 3.02 ± 0.05 g/kg. In our results, the organ weights showed no significant intergroup differences. The white adipose tissue (WAT) weight in group H was significantly higher than that in group N (*p* < 0.05), and the FEO-inhaled groups (H-LFI and H-HFI) had a significantly higher weight of WAT than group H (*p* < 0.05). Regardless of the type of food intake, the weights of brown adipose tissue (BAT) were not significantly different between groups N and H, while group LFI significantly increased compared to group H (*p* < 0.05). Lung weights were not significantly different between groups N and H, while the FEO-inhaled groups were significantly higher than those in group H (*p* < 0.05). Adrenal gland weights were not significantly different among the groups. In addition, the weights of the spleen and epididymis showed no significant differences between the normal diet-fed and HFD-fed groups. On the other hand, the testicular weights in group H dramatically decreased compared to group N (*p* < 0.05); however, the FEO-inhaled groups dramatically increased compared to group H (*p* < 0.05).

WAT is a representative index of visceral fat, and thus, its accumulation may cause obesity [3]. Accordingly, FEO-inhaled groups may have decreased body fat through the reduction of WAT (Figure 2f and Table 2). A previous study observed that trans-anethole, which accounted for the concentration of fennel, increased the activation of PPARα, PPARγ, and AMPK, and decreased pAMPK and WAT weight in animal models [23]. In addition, our previous study reported that the reduction of WAT is influenced by the inhalation of essential oil [1], and an earlier study identified that volatile compounds, which are a major compound in essential oils, caused increased activation of PPARα and energy expenditure via olfactory receptor stimulation [6]. According to the results of several studies, trans-anethole, a dominant compound in FEO, may stimulate the olfactory receptor and/or central nervous system in vivo, thereby causing a reduction in WAT [1,4,6,8].

BAT suppresses metabolic syndrome originating from obesity. In addition, it can control the suppression of visceral fat in vivo according to energy expenditure and heat generation [24]. An increase in the activation of BAT can relieve insulin resistance and/or increase body weight, and BAT activation is associated with BAT mass [23]. In this study, the BAT of the H-LFI group was higher than that of the H group (*p* < 0.05). A previous study reported that BAT mass increased via the administration of trans-anethole in vivo in mice and thus the activation of uncoupling protein-1 (UCP1), which is related to heat activation, increased in mitochondria. Accordingly, the increased BAT mass may suppress the body weight due to a reduction in visceral fat mass [23,24]. In the present study, 0.3% dose inhalation of FEO may have been one of the various factors affecting BAT activation through changes in BAT mass. 

Testicles secrete sexual hormones, such as testosterone, and a change in testicular mass is related to sperm production and testosterone secretion. An earlier study reported that testosterone levels, testicular mass, and gonadosomatic index are positively correlated [25]. In addition, Nejabakhsh et al. identified that the HFD-fed group treated with fennel extracts showed a higher tendency to increase testicular mass and sperm number, with a decreased abnormal sperm ratio [26]. Our results showed that the FEO-inhaled groups had dramatically increased testicular mass compared to group H (*p* < 0.05), and showed no significant differences compared to group N. Accordingly, HFD intake may lead to decreased testicular mass, while FEO inhalation may prevent decreased testicular mass. 

### 3.4. Plasma Analysis

Plasma biomarkers were analyzed using an ELISA kit, and the results are shown in Table 2. The total cholesterol (TC) content of the H-HFI group was the highest among various groups (*p* < 0.05), and groups H and H-LFI showed no significant differences compared to group N. The levels of HDL-cholesterol were dramatically increased in group H compared to group N (*p* < 0.05), and the FEO-inhaled groups had the highest HDL-cholesterol compared to group H (*p* < 0.05). On the other hand, the LDL-cholesterol levels were dramatically decreased in group H-LFI compared to group H (*p* < 0.05); however, group H-HFI was significantly increased compared to group H and group N (*p* < 0.05). The atherogenic index (AI) and cardiac risk factor (CRF) showed the highest levels in group N compared to the other groups (*p* < 0.05), and the H-LFI group had the lowest level among the groups (*p* < 0.05). The ratio of LDL-cholesterol to HDL-cholesterol (LHR) was significantly higher in group H than in groups N and H-LFI (*p* < 0.05), while the ratio in group H was lower than that in group H-HFI (*p* < 0.05). Triglyceride (TG) dramatically increased in group H compared to those in group N (*p* < 0.05). Group H-HFI had significantly higher TG levels than group H (*p* < 0.05). On the other hand, group H-LFI had significantly decreased TG levels than group H (*p* < 0.05), and there was no significant difference between the H-LFI and N groups. Cortisol and leptin levels showed no significant differences among all groups. The levels of insulin and insulin resistance (HOMA-IR) of group N were higher than those of the other groups (*p* < 0.05), and the FEO-inhaled groups were significantly lower than those of the H group (*p* < 0.05). The levels of testosterone were found to be relatively lower in group H than in group N, while the H-LFI group was significantly higher than groups N and H (*p* < 0.05). ALT displayed no significant intergroup differences, while AST displayed a significant increase, as observed in group H compared to group N, as well as FEO-inhaled groups (*p* < 0.05). However, AST displayed no significant differences between the N-and FEO-inhaled groups. 

The concentrations of cholesterol and TG are important indicators of cardiovascular health [3]. Cholesterol is separated by TC, LDL-cholesterol, and HDL-cholesterol, and then increased LDL-cholesterol and decreased HDL-cholesterol may induce the prevalence of cardiovascular diseases, such as disorders of blood pressure and cardiovascular health in vivo [3]. In this study, the HFD-fed groups showed increased HDL-cholesterol levels. On the other hand, LDL-cholesterol levels were decreased in group H-LFI compared to group H; however, group H-HFI showed increased HDL-cholesterol levels compared to group H. Increased HDL-cholesterol levels may be influenced by an increase in HFD intake and the FEO inhalation concentration (Table 2). TG showed a decreasing effect on the inhalation of 1% FEO. A previous study reported that HDL-cholesterol decreased as the PEO inhalation concentration increased, and inhalation of PEO decreased LDL-cholesterol. Furthermore, the inhalation of 0.3% PEO decreased the accumulation of WAT; however, the inhalation of 1% PEO increased the accumulation of WAT [1]. Another previous study reported that trans-anethole, which is the main compound of fennel, decreased TG levels. According to the above-mentioned results, the inhalation of 1% PEO may show the side-effect of fat accumulation via disorders of CNS and ANS activation, such as hormone regulation and energy storage in vivo [1]. According to the present study, the inhalation of 0.3% FEO may be considered optimal for the effect of decreased LDL-cholesterol versus the inhalation of 1% FEO. Therefore, inhalation of 0.3% FEO may be considered to have positive effects on cardiovascular health, while inhalation of 1% FEO may be considered to cause negative effects, such as increasing arteriosclerosis and blood pressure [1,3]. 

Insulin is a hormone that regulates blood glucose and energy storage and is affected by the activation of the CNS and ANS [4]. In the present study, FEO inhalation caused a decrease in insulin as well as insulin resistance levels. The levels of insulin and insulin resistance are influenced by the accumulation of WAT, and the levels of insulin can affect the secretion of insulin-like growth factor-1 (IGF-1) [1,4]. IGF-1 is quite similar to insulin in its amino acid structure (50%); thus, the secretion and/or level of insulin in vivo may induce the synthesis or secretion of IGF-1. Furthermore, IGF-1 is a growth hormone (GH), and increased secretion of IGF-1 can increase its length gain [1]. Our results showed that the intake of HFD increased insulin levels, insulin resistance, and length. Group H showed the highest level of insulin; however, group H had a lower length gain than the FEO-inhaled groups. The FEO-inhaled groups showed lower insulin resistance than group H. A previous study showed that body weight, body fat, and insulin levels were decreased by aerobic exercise, as well as increased GH and IGF-1 levels [27]. According to a previous study and the results of this study, insulin secretion and insulin resistance may indirectly induce an increased length gain, while increasing insulin resistance, which is affected by the excessive secretion of insulin, is one of the inhibitory factors of growth [1,27].

Testosterone, which is secreted in the testicles, is an androgen-type steroid hormone that may affect the growth of bones, muscles, and skin, as well as the generation of prostaglandins and sperms [26,28]. The present study showed that HFD-fed groups had decreased levels of testosterone, and the inhalation of 1% FEO showed increased testosterone levels compared to group H (*p* < 0.05). Erdemir et al. reported that the levels of testosterone were negatively correlated with HFD intake but positively correlated with testicular weight [28]. In addition, the testicular weight increases by the intake of fennel extracts [26]. Therefore, FEO inhalation may also influence the change in testicular weight, and may prevent the decreased secretion of testosterone according to the prevalence of obesity [26,28]. 

The ASL and ALT enzymes are associated with hepatotoxicity, and increasing hepatotoxicity can induce a negative effect on liver health by causing diseases, such as hepatocirrhosis and liver cancer [1]. Inhalation of FEO showed no significant variation in ALT levels among all groups. On the other hand, the AST levels decreased in the FEO-inhaled groups compared to group H (*p* < 0.05), and there has been no significant negative effect of hepatotoxicity, and it may induce the suppression of hepatotoxicity according to the decreased AST levels [11,29].

### 3.5. Blood Glucose

Blood glucose levels were measured using a blood glucose meter and are presented in Table 3. In the initial period, the blood glucose levels showed no significant differences among all groups, while the FEO-inhaled groups (seventh week) were dramatically decreased compared to group H (seventh week). In the final period, the levels of glucose showed no significant differences among the groups; thus, the short-term effects of decreased blood glucose levels were identified via FEO inhalation.

Blood glucose levels play an important role in evaluating diabetes, and increased levels of blood glucose may cause hyperglycemia [26,29]. An earlier study observed that blood glucose levels were decreased by an intake of fennel extracts [30]; however, we identified only short-term decreasing effects via FEO inhalation.

### 3.6. Blood Pressure

Blood pressure, which is composed of systolic blood pressure (SBP), diastolic blood pressure (DBP), and pulse was measured, and the results are presented in Table 4. In the initial period, SBP showed no significant differences among all groups, while SBP, which was measured at the 12th week, was significantly decreased in the FEO-inhaled groups (H-LFI and H-HFI) compared to group H (*p* < 0.05). In particular, the H-LFI group had a significantly lower SBP than the N group (*p* < 0.05). DBP and pulse, which were measured in the first week, were not significantly different among the groups. In the final period, the pulse of the FEO-inhaled groups was significantly lower than that of group H (*p* < 0.05); however, DBP showed no significant intergroup differences.

Hypertension is caused by the accumulation of visceral fat and/or increased body weight, as well as increased levels of insulin, blood glucose, visceral mass, and/or decreased HDL cholesterol [1,3]. In the present study, the levels of insulin, insulin resistance, and visceral fat mass were decreased, and HDL-cholesterol was increased via FEO inhalation. In addition, SBP and pulse, which were measured at the 12th week, were simultaneously dramatically decreased in the FEO inhaled groups compared to group H (*p* < 0.05). Increased HDL cholesterol may play an important role in lowering blood pressure according to the control of the CNS and ANS (e.g., sympathetic and parasympathetic nerves), and visceral fat mass is positively correlated with insulin resistance and blood pressure [1,3]. In addition, fennel is known to suppress cardiovascular diseases (e.g., hypertension, atherosclerosis, and hyperlipidemia), and several previous studies have reported that fennel extracts prevent increasing SBP and pulse [26,29,30]. Moreover, Hong et al. reported that SBP was decreased via inhalation of volatile compounds in PEO, and various studies have identified the blood-pressure-lowering effects of inhalation of volatiles [1,20,22]. In this study, the H-LFI and H-HFI groups had decreased SBP and/or pulse via FEO inhalation; thus, FEO volatiles may also influence decreased cardiovascular diseases. In general, most in vivo experiments have conducted oral administration. However, the animal study, which investigated the physiological effects on inhalation of volatile compounds, is insufficient. Therefore, a comparative study for advantages and side effects of FEO inhalation versus oral administration has not existed. To date, hepatotoxicity of essential oil via oral administration and recommended oral doses for toxicological safety have been represented [31]. Meanwhile, there is currently no research about the hepatoxicity of essential oil inhalation.

## 4. Conclusions

In conclusion, this research indicates that inhalation of FEO protected lipid and metabolic dysfunction in high-fat diet-induced obese rats. Therefore, these advantages suggest that the inhalation of FEO could lead to significant metabolic health gains and improvement of diet-induced obesity. Furthermore, these findings may contribute to the initial design of future studies.

## Figures and Tables

**Table 1:** Odor active compounds (OACs) of fennel essential oil by gas chromatography/mass spectrometry (GC/MS) and GC-olfactometry (GC-O).

Major Compound	RT ^a^	RI ^b^	Mean ± SD	Mean ± SD	Odor Intensity	Odor Description	I.D. ^c^
	(min)		(μg/100 g)	(%)			
Aldehyde (1)							
*p*-Anisaldehyde	22.57	1276	304.17 ± 38.79	1.18 ± 0.15	1	Fennel	MS ^**d**^/RI
Hydrocarbons (5)							
*α*-Pinene	13.03	961	1635.87 ± 139.95	6.33 ± 0.54	1	Herb	MS/RI
Limonene	16.11	1056	733.77 ± 18.60	2.84 ± 0.07	1	Fennel	MS/RI
Estragole	21.16	1224	1556.21 ± 40.28	6.02 ± 0.15	1	Fennel	MS/RI
*cis*-Anethole	22.55	1275	382.86 ± 35.95	1.48 ± 0.16	2	Fennel	MS
*trans*-Anethole	23.69	1317	13,100.47 ± 3971.88	50.69 ± 15.37	2	Fennel	MS
Ketone (1)							
Fenchone	18.23	1123	4956.46 ± 117.31	19.18 ± 4.32	1	Herb, fragrance	MS

Data are given as mean ± SD values from experiments performed in duplicate. ^a^ RT: retention time; ^b^ RI: retention index; ^c^ I.D.: identification; ^d^ MS: mass spectrum.

**Table 2:** Effects of fennel essential oil inhalation on growth parameters, organ weights, and plasma biomarkers in male rats fed a normal diet and high-fat diet (HFD).

Parameters	N	H	H-LFI	H-HFI
Growth parameters				
Food intake (g/day)	18.03 ± 0.70 ^a1^	15.09 ± 0.64 ^b^	14.92 ± 0.37 ^b^	15.21 ± 0.78 ^b^
Initial length (cm)	17.3 ± 0.3 ^a^	16.7 ± 0.1 ^b^	16.7 ± 0.1 ^b^	16.5 ± 0.2 ^b^
Final length (cm)	23.4 ± 0.3 ^b^	23.4 ± 0.1 ^b^	24.1 ± 0.1 ^a^	23.9 ± 0.1 ^a^
Length gain (cm)	6.1 ± 0.2 ^c^	6.6 ± 0.1 ^b^	7.4 ± 0.1 ^a^	7.4 ± 0.2 ^a^
Energy intake (kcal/day)	50.48 ± 1.93 ^b^	69.40 ± 2.94 ^a^	68.64 ± 1.68 ^a^	69.95 ± 3.58 ^a^
BMI	7.71 ± 0.45 ^b^	9.17 ± 0.30 ^a^	8.13 ± 0.13 ^b^	7.92 ± 0.15 ^b^
FER (%)	17.65 ± 1.29 ^b^	26.93 ± 1.83 ^a^	25.45 ± 1.48 ^a^	23.27 ± 1.49 ^a^
Organ weights				
Brain (g/kg)	3.91 ± 0.17 ^a^	3.57 ± 0.25 ^a^	3.94 ± 0.06 ^a^	3.98 ± 0.39 ^a^
Liver (g/kg)	24.84 ± 0.29 ^a^	24.81 ± 5.46 ^a^	24.44 ± 0.64 ^a^	25.41 ± 2.28 ^a^
Kidney (g/kg)	5.71 ± 0.18 ^a^	4.46 ± 0.11 ^b^	5.46 ± 0.14 ^ab^	5.36 ± 0.72 ^ab^
Heart (g/kg)	3.02 ± 0.05 ^a^	2.34 ± 0.12 ^b^	3.01 ± 0.10 ^a^	2.89 ± 0.47 ^ab^
WAT (g/kg)	26.76 ± 1.71 ^b^	41.37 ± 1.64 ^a^	32.22 ± 4.51 ^b^	29.30 ± 1.06 ^b^
BAT (g/kg)	0.53 ± 0.04 ^b^	0.62 ± 0.07 ^b^	1.04 ± 0.06 ^a^	0.55 ± 0.08 ^b^
Lung (g/kg)	3.45 ± 0.11 ^ab^	3.10 ± 0.33 ^b^	3.87 ± 0.06 ^a^	3.89 ± 0.32 ^a^
Adrenal galnds(g/kg)	0.12 ± 0.01 ^b^	0.12 ± 0.01 ^b^	0.13 ± 0.01 ^b^	0.15 ± 0.01 ^b^
Spleen (g/kg)	1.67 ± 0.01 ^a^	1.34 ± 0.03 ^b^	1.47 ± 0.19 ^ab^	1.75 ± 0.14 ^a^
Testicular (g/kg)	9.81 ± 0.57 ^a^	7.52 ± 0.45 ^b^	9.30 ± 0.17 ^a^	9.53 ± 0.48 ^a^
Epididymis (g/kg)	2.87 ± 0.12 ^a^	2.59 ± 0.20 ^a^	2.89 ± 0.07 ^a^	2.70 ± 0.26 ^a^
Plasma biomarkers				
TC (mg/dL)	128.91 ± 3.63 ^b^	131.45 ± 2.07 ^b^	124.07 ± 4.05 ^b^	173.44 ± 1.77 ^a^
HDL (mg/dL)	35.50 ± 0.79 ^d^	42.51 ± 0.44 ^c^	49.90 ± 1.13 ^b^	52.82 ± 0.25 ^a^
LDL (mg/dL)	46.72 ± 1.78 ^c^	75.62 ± 1.59 ^b^	55.95 ± 5.41 ^c^	109.85 ± 5.63 ^a^
AI (mg/dL)	2.61 ± 0.05 ^a^	2.10 ± 0.10 ^c^	1.44 ± 0.04 ^d^	2.27 ± 0.04 ^b^
CRF (mg/dL)	3.61 ± 0.05 ^a^	3.17 ± 0.10 ^b^	2.44 ± 0.04 ^c^	3.27 ± 0.04 ^b^
LHR (mg/dL)	131.70 ± 7.94 ^c^	177.92 ± 5.61 ^b^	112.05 ± 8.29 ^c^	207.93 ± 9.69 ^a^
TG (mg/dL)	94.20 ± 3.03 ^b^	102.52 ± 3.14 ^a^	88.75 ± 2.02 ^b^	111.91 ± 6.61 ^a^
Cortisol (ng/dL)	4.29 ± 0.54 ^a^	4.38 ± 0.37 ^a^	4.50 ± 0.05 ^a^	4.07 ± 0.07 ^a^
Insulin (ng/mL)	0.42 ± 0.17 ^d^	4.13 ± 0.14 ^a^	1.79 ± 0.13 ^c^	2.83 ± 0.04 ^b^
HOMA-IR	2.19 ± 1.10 ^d^	22.57 ± 2.78 ^a^	8.54 ± 1.25 ^c^	14.05 ± 0.39 ^b^
Leptin (pg/mL)	2842.79 ± 173.45 ^a^	3926.00 ± 225.35 ^a^	3863.55 ± 790.87 ^a^	2711.10 ± 936.96 ^a^
Testosterone (pg/mL)	0.43 ± 0.11 ^b^	0.29 ± 0.07 ^b^	1.38 ± 0.19 ^a^	0.31 ± 0.01 ^b^
ALT (Karmen/mL)	5.16 ± 2.71 ^a^	8.29 ± 3.73 ^a^	10.25 ± 8.53 ^a^	3.05 ± 0.79 ^a^
AST (Karmen/mL)	40.00 ± 24.28 ^a^	70.63 ± 1.36 ^aA2^	54.08 ± 7.54 ^aB^	56.46 ± 1.36 ^aB^

Data are given as mean ± SD values from experiments performed in triplicate. ^1^ Means with different small letters (a–d) correspond to the significant differences determined among all groups using the non-parametric Friedman test followed by Dunn’s test (*p* < 0.05). ^2^ Means with different capital letters (A and B) correspond to the significant differences determined among all HFD groups through the non-parametric Friedman test followed by Dunn’s test (*p* < 0.05). N: normal diet-induced control, H: high-fat diet-induced control, H-LFI: high-fat diet-induced and 0.3% FEO-inhaled rats, H-HF: high-fat diet-induced and 1% FEO-inhaled rats. BMI (Body mass index) = Body weight/Length^2^. FER (Food efficiency ratio) = Body weight gain/food intake × 100. AI (Atherogenic index) = (TC-HDL/HDL). CRF (Cardiac risk factor) = (TC/HDL). LHR = LDL/HDL × 100. HOMA-IR (Insulin resistance) = Insulin × Blood glucose/405.

**Table 3:** Effect of fennel essential oil inhalation on blood glucose.

Blood Glucose	Weak		
(mg/dL)	Initial Period	Obesity Induced Period	Final Period
N	102.3 ± 7.2 ^a1^	108.7 ± 5.5 ^b^	114.0 ± 12.5 ^a^
H	108.0 ± 3.6 ^a^	126.7 ± 6.5 ^a^	122.7 ± 11.0 ^a^
H-LFI	101.7 ± 8.1 ^a^	107.2 ± 6.6 ^b^	117.6 ± 8.0 ^a^
H-HFI	112.3 ± 3.8 ^a^	109.7 ± 3.1 ^b^	111.7 ± 1.5 ^a^

Data are expressed as mean ± SD values from experiments performed in triplicate. ^1^ Means with different small letters (a–b) correspond to the significant differences determined among all groups using the non-parametric Friedman test, followed by Dunn’s test (*p* < 0.05). Obesity-induced period: 7 weeks.

**Table 4:** Effect of fennel essential oil inhalation on the systolic blood pressure, diastolic blood pressure, and pulse.

Systolic(mmHg)	Weak	
	Initial Period	Final Period
N	154.7 ± 3.2 ^a1^	199.3 ± 3.8 ^bc^
H	153.0 ± 5.3 ^a^	215.0 ± 7.0 ^a^
H-LFI	157.3 ± 12.4 ^a^	190.0 ± 2.0 ^c^
H-HFI	148.7 ± 4.9 ^a^	201.0 ± 1.0 ^b^
Diastolic(mmHg)		
N	69.7 ± 1.5 ^a^	133.3 ± 12.1 ^a^
H	52.0 ± 5.3 ^a^	114.0 ± 4.0 ^ab^
H-LFI	60.3 ± 10.1 ^a^	131.3 ± 5.5 ^ab^
H-HFI	55.0 ± 19.5 ^a^	113.3 ± 5.5 ^b^
Pulse(beats/min)		
N	422.0 ± 13.1 ^a^	377.0 ± 14.9 ^b^
H	415.7 ± 18.6 ^a^	415.3 ± 8.3 ^aA2^
H-LFI	408.7 ± 8.1 ^a^	381.3 ± 7.44 ^bB^
H-HFI	424.0 ± 22.7 ^a^	382.3 ± 6.4 ^abB^

Data are expressed as mean ± SD values from experiments performed in triplicate. ^1^ Means with different small letters (a–c) correspond to the significant differences determined among all groups using the non-parametric Friedman test followed by Dunn’s test (*p* < 0.05). ^2^ Means with different capital letters (A and B) correspond to the significant differences determined among all HFD groups through the non-parametric Friedman test followed by Dunn’s test (*p* < 0.05).

## Data Availability

The data presented in this study are available.
